# A commentary on ‘Regional differences in bone mineral density biomechanically induce a higher risk of adjacent vertebral fracture after percutaneous vertebroplasty’

**DOI:** 10.1097/JS9.0000000000001418

**Published:** 2024-04-03

**Authors:** Chuanliang Chen, Xiaochong Zou, Mingyang Jiang, Ke Zhang, Ruqiong Wei

**Affiliations:** a Department of Bone and Joint Surgery, The First Affiliated Hospital of Guangxi Medical University; b Department of Rehabilitation Medicine, The First Affiliated Hospital of Guangxi Medical University, Nanning, People’s Republic of China

*Dear Editor,*

It was with great interest that we read the article published in the *International Journal of Surgery*. Li *et al*.^1^ conducted a retrospective study on osteoporotic vertebral compressive fracture patients treated with percutaneous vertebroplasty (PVP). The results demonstrated that following PVP surgery, the local biomechanical environment deteriorates and the aggravation of regional bone mineral density (BMD) disparities increases the risk of adjacent vertebral fracture (AVF).

In osteoporotic vertebral compressive fracture patients getting PVP, AVF appeared to be a common and serious complication that led to poor long-term results and the recurrence of pain-related complaints. Numerous researchers thus concentrated on its causes and associated risk factors. Low BMD, disc degeneration, asymmetric cement distribution, excessive cement volume, and regional kyphosis were found to be risk factors of AVF following PVP in their investigations^2^. However, its mechanism is still unknown. It was concluded that the primary cause of AVF following PVP may be the decrease in mechanical absorption capacity and spinal stiffness.

Researchers have shown a strong correlation between biomechanics and postoperative complications of PVP^3^. Studies have indicated that the distribution of bone cement and the rigidity of the vertebral body, which impact stress balance on the latter, are significant influencing variables for the recompression of the PVP-operated vertebrae. It appears that the unbalanced distribution of bone cement may weaken the non-augmented side’s physical strength and increase the risk of fracture recurrence. In the meantime, bilateral percutaneous vertebroplasty (BPVP) would create a balanced load transfer with the rigidity and symmetrical distribution of bone cement, which might lessen the strain on nearby vertebral bodies and lower the chance of future fractures^4^.

This study compares the geographical differences between patients with and without AVF in the BMD of the vertebral body next to the PVP section. They used a biomimetic lumbar finite element model to confirm the biomechanical importance of the exacerbation of these BMD disparities. However, there were still a lot of restrictions. Firstly, to some extent, there may be selection bias in this retrospective study. Secondly, the lack of some clinical characteristics, such as a history of illness, led to bias. It has been claimed that risk factors for new VCF after PVP were age, gender, hounsfield unit value, thoracolumbar junction fracture, cement leakage, zoledronic acid, and previous fracture history^5^. Although some of those remain controversial; however, only the year, gender, and BMI were considered as demographic covariates in this article. Thirdly, it’s possible that some residual confounding variables were overlooked. PVP can be divided into unilateral percutaneous vertebroplasty and BPVP. Studies have shown that BPVP could balance the stress on the vertebral body, reduce the maximum stress on the intervertebral disc, and offer advantages in terms of stability compared with unilateral percutaneous vertebroplasty.

Large sample size prospective multicenter randomized controlled trials are still required to more thoroughly examine the risk factors of developing VCF following PVP in the future. If more cases are added in the future, subgroup analysis and long-term follow-up will be necessary. Thus, future biomechanical assessments should construct finite element models that can more correctly reflect the human situation and create a personalized vertebroplasty plan for each patient based on the current work and general experiences with datasets of numerous individuals. In the future, in-vitro biomechanical tests and clinical investigations will be carried out to assess the results of this investigation.

## Ethical approval

Not applicable.

## Consent

Not applicable.

## Sources of funding

Not applicable.

## Author contribution

C.C. and X.Z.: study concept or design, data collection, data analysis or interpretation, and writing the paper; M.J. and K.Z.: data collection; R.W.: study concept or design and writing and revising the paper.

## Conflicts of interest disclosure

The authors have no conflicts of interest to disclose.

## Research registration unique identifying number (UIN)

Not applicable.

## Guarantor

Ruqiong Wei.

## Data availability statement

This manuscript is a comment. Don’t need a Data availability statement. However, all the data from the current study are publicly available.

## Provenance and peer review

Not applicable.
